# Draft Genome Sequences of Two Multidrug-Resistant Extended-Spectrum-β-Lactamase-Producing *Klebsiella pneumoniae* Strains Causing Bloodstream Infections

**DOI:** 10.1128/genomeA.01533-15

**Published:** 2016-01-21

**Authors:** Eran Carasso, Mali Salmon-Divon, Yehuda Carmeli, Ehud Banin, Shiri Navon-Venezia

**Affiliations:** a Department of Molecular Biology, Ariel University, Ariel, Israel; b The Mina and Everard Goodman Faculty of Life Sciences, The Institute for Nanotechnology and Advanced Materials, Bar-Ilan University, Ramat-Gan, Israel; c Tel Aviv Medical Center, Division of Epidemiology, Sackler School of Medicine, Tel Aviv University, Tel Aviv, Israel

## Abstract

Multidrug-resistant (MDR) *Klebsiella pneumoniae* has become a major contributor to nosocomial bloodstream infections. Here, we report the draft genome sequences of two MDR extended-spectrum-β-lactamase-producing strains causing bloodstream infections. These sequenced genomes display a wide-spectrum virulence arsenal and will help us understand the genomic basis of *K. pneumoniae* virulence.

## GENOME ANNOUNCEMENT

Bacterial bloodstream infections (BSIs) are considered to be life-threatening infections in humans (1). *Klebsiella pneumoniae* is one of the major Gram-negative pathogens (2) that has become a major contributor to nosocomial BSIs. With the increasing problem of antibiotic resistance and specifically the dissemination of extended-spectrum β-lactamases (ESBLs), the prevalence of ESBL-producing *K. pneumoniae* strains is escalating worldwide. ESBLs are plasmid-mediated enzymes that confer resistance to all oxyimino-cephalosporins and aztreonam antibiotics (3), and they often cause bacteria to exhibit a multidrug-resistant (MDR) phenotype (4). The prevalence of MDR *K. pneumoniae* causing BSIs is dramatically rising and may reach up to two-thirds of all *K. pneumoniae* BSIs (5, 6); MDR is associated with increased patient morbidity and mortality (7) and prolonged hospitalization and costs, irrespective of the underlying illness (8). However, despite this critical concern, little is known about the association between ESBL production and pathogenesis in *K. pneumoniae*, and there is a need for additional genetic data to further elucidate this association.

ESBL-producing *K. pneumoniae* strains B199 and B86 were isolated from blood cultures from 2 patients with BSIs at the Tel Aviv Medical Center, Tel Aviv, Israel. Genomic DNA was purified using the DNeasy blood and tissue kit (Qiagen). Library preparation was done using the Nextera XT DNA sample preparation kit (Illumina). Whole-genome paired-end sequencing was performed using the MiSeq sequencer (Illumina) at the Technion Genome Center (Haifa, Israel). A total of 2,449,738 and 2,140,594 paired-end reads were generated for strains B199 and B86, respectively, at a read length of 250 bp, with an average coverage of approximately 170×. Any adapter contamination was removed using the sequencer’s built-in read trimming tool, and low-quality bases were removed from the ends of the reads using FastQC (9).

Genome assembly was performed using A5-miseq pipeline (10), with default parameters, into 88 (*N*_50_, 251,281 bp) and 53 (*N*_50_, 266,552 bp) contigs for *K. pneumoniae* strains B199 and B86, respectively. The final draft genome sequences consist of a combined 5,748,444 and 5,675,784 bp, with 56.7% and 57% G+C content, for B199 and B86, respectively. Contig annotation was carried out using the Rapid Annotations using Subsystems Technology (RAST) server (11). The final draft genomes of strains B199 and B86 contain, respectively, 5,451 and 5,418 coding sequences (of which 4,452 and 4,463 coding sequences possess annotated functions, with the remaining being hypothetical). One hundred twenty-four and 110 RNA genes (of which 88 and 80 are tRNAs) were detected in B199 and B86, respectively.

Genome analysis of the two *K. pneumoniae* strains revealed multiple antibiotic and metal resistance genes, including *bla*_CTX-M_ ESBLs (*bla*_CTX-M-2_ in B199 and *bla*_CTX-M-15_ in B86), fluoroquinolones, and fosfomycin. Resistance to the aminoglycosides was present in strain B199 only. Both strains contained multiple resistances to metals (copper, cobalt, zinc, cadmium, arsenic, and chromium), as well as multidrug resistance efflux pumps (*mdtABCD*, multiple antibiotic resistance [MAR], and tripartite systems). These data expand our knowledge on MDR and virulence in this pathogen.

### Nucleotide sequence accession numbers.

This whole-genome shotgun project has been deposited in DDBJ/EMBL/GenBank under the accession numbers LJCB00000000 and LJCD00000000 for *K. pneumoniae* B199 and B86, respectively.
